# Prevalence of *Schistosoma* mono- and co-infections with multiple common parasites and associated risk factors and morbidity profile among adults in the Taabo health and demographic surveillance system, South-Central Côte d’Ivoire

**DOI:** 10.1186/s40249-021-00925-1

**Published:** 2022-01-05

**Authors:** Fidèle K. Bassa, Ikenna C. Eze, Rufin K. Assaré, Clémence Essé, Siaka Koné, Félix Acka, Véronique Laubhouet-Koffi, Dinard Kouassi, Bassirou Bonfoh, Jürg Utzinger, Eliézer K. N’Goran

**Affiliations:** 1 Unité de Formation et de Recherche Biosciences, Université Félix Houphouët-Boigny, 22, P.O. Box 582, Abidjan 22, Côte d’Ivoire; 2 Swiss Tropical and Public Health Institute, 4002 Basel, Switzerland; 3 University of Basel, 4003 Basel, Switzerland; 4 Centre Suisse de Recherches Scientifiques en Côte d’Ivoire, 01, P.O. Box 1303, Abidjan 01, Côte d’Ivoire; 5 Institut d’Ethnosociologie, Université Félix Houphouët-Boigny, 01, P.O. Box 34,, Abidjan 01, Côte d’Ivoire; 6 Institut National de Santé Publique, 01, P.O. Box 47, Abidjan 01, Côte d’Ivoire; 7 Ligue Ivoirienne Contre l’Hypertension Artérielle et les Maladies Cardiovasculaires, 17, P.O. Box 773, Abidjan 17, Côte d’Ivoire

**Keywords:** Adults, Côte d’Ivoire, Morbidity patterns, Prevalence, Risk factor, *Schistosoma* infection

## Abstract

**Background:**

Schistosomiasis remains an important public health problem, also among adults, and infected individuals not treated serve as a reservoir for continued transmission. Despite this fact, evidence on the epidemiology of schistosomiasis in adults in Côte d’Ivoire is scanty. This study aimed to determine the prevalence and risk factors of *Schistosoma* infection and co-infection with other helminth species and *Plasmodium* among adults in the Taabo region in the south-central part of Côte d’Ivoire.

**Methods:**

A cross-sectional survey was carried out in April and May 2017 in the frame of the “Côte d’Ivoire Dual Burden of Disease Study” (CoDuBu). A total of 901 randomly selected individuals, aged 18–90 years, provided blood, stool and urine samples for the diagnosis of malaria and helminth infections. Stool samples were subjected to the Kato-Katz technique for detection of *Schistosoma mansoni* and soil-transmitted helminth eggs, while urine samples were examined for eggs of *Schistosoma haematobium* and circulating cathodic antigen of *S. mansoni*. Risk factors and morbidity profiles were assessed using health examination and questionnaires. Multinomial logistic regressions were employed to identify risk factors and morbidity patterns associated with *S. mansoni* mono- and co-infections.

**Results:**

The prevalence of *S. mansoni* and *S. haematobium* was 23.2% and 1.0%, respectively. Most *S. mansoni* were mono-infections (81.3%). Independent determinants of *S. mansoni* infection were young age, low socioeconomic status (mono- and co-infection) and poor hygiene practices (co-infection) (*P* < 0.05). *S. mansoni* infection was independently associated with higher pain and symptom scores (mono-infection), poor self-rated health and low healthcare use (co-infection) (*P* < 0.05).

**Conclusions:**

This study showed that adults represent a substantial reservoir of *S. mansoni*. To sustain schistosomiasis control and improve people’s wellbeing, it is important to expand preventive chemotherapy from school-aged children to adults, coupled with hygiene and health education.

## Background

In Côte d’Ivoire, schistosomiasis remains a considerable public health problem [1–3]. In many regions, health education and hygiene conditions are poor, and hence, there is sustained transmission of schistosomiasis and other helminth infections. Following recommendations put forth by the World Health Organization (WHO) 15 years ago [4], a national schistosomiasis control programme was launched [5]. In the meantime, multiple rounds of preventive chemotherapy have been conducted, using praziquantel against schistosomiasis and albendazole or mebendazole against soil-transmitted helminth infections, primarily targeting school-aged children. Of note, a limitation of this strategy is that parts of the population are excluded, such as preschool-aged children, women of reproductive age and adults more generally. This issue might represent an obstacle to the achievement of the Sustainable Development Goals (SDGs) adopted by the United Nations, calling to end neglected tropical diseases such as schistosomiasis by 2030 [6, 7]. Although school-aged children are at highest risk of schistosomiasis in endemic areas, infection of adults remains an important cause of morbidity and even mortality [8, 9]. Infected adults not treated also serve as a reservoir of the *Schistosoma* parasites. Nonetheless, the epidemiology of schistosomiasis in adults in Côte d’Ivoire is poorly understood [1, 10, 11].

Previous studies focusing on school-aged children showed that *Schistosoma* infection is common in the south-central part of Côte d’Ivoire, particularly the region of Taabo, with *Schistosoma haematobium* being the predominant species [12, 13]. The Bandama River and the hydroelectric dam built across the river in the late 1970s constitute a key feature of this region that governs the transmission of schistosomiasis [14]. However, in 2011, as a result of an investigation carried out by the Taabo Health and Demographic System (HDSS), multiple cases of intestinal schistosomiasis were observed for the first time downstream of the dam. Interestingly, a recent cross-sectional epidemiological study suggested a shift from *S. haematobium* to *S. mansoni* [11]. Moreover, *Schistosoma* infection may occur concurrently with soil-transmitted helminths (STHs) and *Plasmodium* infections [15, 16]. In this context, risk factors for *Schistosoma* infection and co-infection with these diverse parasites and related morbidity may overlap extensively. Yet, there are limited data on adults.

Against this background, it is important to characterize the epidemiological profile of this important schistosomiasis focus, which will shape the design, implementation and monitoring of a control strategy that is tailored to the social-ecological setting. The objectives of this study were to determine the prevalence of *Schistosoma* infection and co-infection with other helminth species and *Plasmodium*, as well as to identify associated risk factors and morbidities among adults in the Taabo region, Côte d’Ivoire.

## Methods

### Study area and participants

This study was conducted in the Taabo HDSS, located in the south-central part of Côte d’Ivoire. The Taabo HDSS, launched in late 2008, covers a surface area of 980 km^2^ and comprises of a small town (Taabo-Cité), 13 main villages and over 100 hamlets with an initial population of approximately 38,000 people. It provides high-quality longitudinal data on all the residents, with particular emphasis on demography (age, sex, education, pregnancy, birth, causes of death, migration, ethnicity, nationality, religion and employment). Data are collected prospectively at the individual and household level, usually done in three rounds per year [17]. The Taabo HDSS serves also as a platform for specific epidemiological studies like the one presented here and, under the leadership of district health personnel with support of the Taabo HDSS staff, interventions were undertaken for specific parasitic infections. The strong link with the health system not only enables effective interventions, but also facilitates transfer of key results to health authorities. The Taabo HDSS is predominantly rural and most income generating activities are related to cash crop cultivation (cocoa and coffee) and fishing. The health system comprises primary health care facilities in the villages and a small general hospital in Taabo-Cité.

Investigations were carried out within the frame of the “Côte d’Ivoire Dual Burden of Disease” (CoDuBu) study, aiming for a deeper understanding of interrelation of common infectious diseases and non-communicable diseases among adults in the Taabo HDSS. The design of the CoDuBu study is described in detail elsewhere [18]. In brief, for this multi-disease, co-morbidity study, a sample size of 976 adults aged ≥ 18 years was estimated based on an expected malaria and diabetes co-occurrence prevalence of 2%, an error margin of 1%, a 95% confidence level and a nonresponse rate of 30% for a reference population of 200,000. Using a sampling frame generated by the Taabo HDSS, participants were randomly selected from three sites: Tokohiri, Amani-Ménou and Taabo-Cité. These sites are located along the Bandama River and are illustrative of the diverse residence settings encountered in the Taabo HDSS: Taabo-Cité reflecting the only semi-urban setting that is situated in close proximity to the man-made dam and the impounded lake, while Tokohiri and Amani-Ménou are two villages located upstream and downstream of the man-made dam, respectively.

### Ethical considerations

The CoDuBu study adheres to the principles of Declarations of Helsinki. Ethical approvals were obtained from the National Ethics Committee for Life and Health Sciences of Côte d’Ivoire (reference no. 032/IMSHP/CNER-kp; date of approval: March 24, 2017) and the Ethics Committee of North-West and Central Switzerland (reference no. 2016-00143; date of approval: May 2, 2016). Before the implementation of field activities, the objectives, procedures, potential risks and benefits of the study were explained to community leaders, and local approvals were also obtained. All participants provided written informed consent prior to enrollment in the study. Participants who were diagnosed with any screened infection were treated free of charge, according to national guidelines.

### Data collection and administration of questionnaire

A cross-sectional survey was carried out in April and May 2017. Randomly selected adults who agreed to participate in the study received prelabelled containers to be partially filled with their fresh morning stool and mid-stream urine samples the next day. Stool and urine samples were collected at home and transferred in cool boxes to the Taabo-Cité laboratory for analysis. The same day, participants were invited at the local health centre to provide venous whole blood samples in ethylenediaminetetraacetic (EDTA) tubes, which were taken for laboratory screening.

Participants had anthropometric (including body weight and height) and body temperature measurements recorded. Body weight (nearest 0.1 kg) and height (nearest 1 cm) were measured using SECA weighing scales and stadiometers (SECA GmbH; Hamburg, Germany), respectively. Body temperature (nearest 0.1 °C) was measured using Omron auricular thermometers (Omron Healthcare; Kyoto, Japan).

Participants also had interviews covering lifestyle and health-related characteristics. Demographic, socioeconomic, water, sanitation and hygiene (WASH) and health-related indicators were collected using a combination of the CoDuBu study and the Taabo HDSS questionnaires. Demographic indicators collected were age, sex and area of residency. For socioeconomic status, indicators were education, wealth index and occupation. WASH indicators were assessed by documenting seven parameters: (1) presence of household toilet; (2) use of surface water; (3) water storage; (4) disposal of household waste; (5) disposal of toilet water in the open; (6) faecal handling; and (7) handwashing practices. Health-related indicators were clinical symptoms, self-rated health and healthcare use in the preceding 12 months. Clinical symptoms included 10 parameters: (1) abdominal pain; (2) general body pain; (3) use of pain medications; (4) pain disturbance; (5) fever; (6) fatigue; (7) nausea or vomiting; (8) diarrhea; (9) blood in the stool; and (10) blood in the urine. Pain comprised general body pain, pain severity, abdominal pain and pain medication. Self-reported fever and presence of blood in the urine were augmented with objectively measured body temperature (> 37.5 °C) and positive dipstick test for haematuria, respectively.

### Laboratory procedures

Stool samples were examined using the Baermann technique for the diagnosis of *Strongyloides stercoralis* [19] and the Kato-Katz technique [20] for *S. mansoni* and soil-transmitted helminths. Duplicate Kato-Katz thick smears were prepared from each stool sample. Within an hour after preparation, the Kato-Katz thick smears were examined under a microscope by experienced laboratory technicians for the diagnosis of hookworm eggs as the eggs deteriorate rapidly. The same thick smears were re-examined later for the diagnosis of the eggs of *S. mansoni*, *Ascaris lumbricoides* and *Trichuris trichiura*.

Urine samples were examined for the presence of *S. haematobium* eggs using a filtration method [21], for *S. mansoni* infection using a point-of-care circulating cathodic antigen (POC-CCA) cassette test (ICT Diagnostics; Cape Town, South Africa) and for microhaematuria using a Roche Combur-10 test (Roche Diagnostics; Basel, Switzerland). In accordance to the manufacturer’s instructions, POC-CCA tests were scored as either negative or positive, the latter stratified into trace, 1 +, 2 + or 3 + .

Blood samples were subjected to a rapid diagnostic test (ICT Diagnostics; Cape Town, South Africa) and thick and thin blood films were examined under a microscope for the assessment of *Plasmodium* infection. Haemoglobin level in blood samples was measured using Hemocue 301 system (Angelholm, Sweden).

### Statistical analysis

Participants were considered as positive for a specific infection if at least one of its diagnostic methods revealed a positive result, and if otherwise, were considered negative. Participants’ age was classified along the median value (≤ 41 years vs > 41 years). Study area was classified into semi-urban (Taabo-Cité) and rural (Amani-Ménou and Tokohiri). Educational status was classified into formal education (i.e., attending primary, secondary or tertiary education institution) and no formal education (i.e., not attending any formal schools). Occupation was classified into farming (i.e., agriculture, fishing) and non-farming occupation. Participants were categorized into three socioeconomic strata (lowest, middle and upper wealth tertiles) based on an index derived from a principal component analysis of their respective household assets (e.g., radio, television, motorcycle, bicycle, cell phone, fan and refrigerator) [17]. In order to do this, assets were coded as binary variables (1 = yes, 0 = no). The first principal component was used to derive wealth tertiles according to a widely used methodology [22], readily adapted to the local context. An additive hygiene score (comprising the seven WASH parameters; range: 0–7) was generated and participants were classified (along the median value) as having either poor or good hygiene practices.

Anaemia was defined as haemoglobin < 12 g/100 ml in females and < 13 g/100 ml in males [23]. Body mass index (BMI) was calculated as a ratio of weight and height-squared and underweight was defined as BMI < 18.5 kg/m^2^ [24]. An additive pain score (comprising the four pain parameters; range: 0–4) was generated, and participants were classified (along the median value) as having either lower or higher pain problems. Similarly, additive general symptom score (comprising the 10 symptoms; range: 0–10) was also generated, and participants were classified (along the median value) as having either lower or higher symptoms. These additive scores represent more robust and minimally biased measures (in comparison to using the single components) to capture to some extent, the hygiene-related risk factors and the morbidity-related parameters in the study sample.

Descriptive statistics were used to summarize characteristics of the study sample. Categorical variables were summarized as counts and proportions, whereas continuous variables were summarized as medians and interquartile ranges. Infection prevalence was compared between subgroups of the study sample and the Pearson’s *χ*^2^ test was used to test the differences between the subgroups.

A bivariate logistic regression analysis based on the crude odds ratio (c*OR*) was performed to identify potential risk factors associated with *S. mansoni* infection status. Significant predictors from bivariate model were included in the multivariate analysis. Adjusted odds ratio (a*OR*) and *P*-value from multivariate logistic regression model were utilized to investigate the strength of associations. The potential risk factors assessed were age (≤ 41 years vs > 41 years), sex (males vs females), study area (semi-urban vs rural), formal education (yes vs no), occupation (farming vs non-farming) and wealth index (lowest vs upper tertiles) and hygiene score (> 3 vs ≤ 3). To reduce the bias (on the regression estimates) that may arise due to other infectious agents, participants who had other infections (*Plasmodium* and soil-transmitted helminths) without *S. mansoni* were excluded from the regression analyses. Thus, participants were stratified by *S. mansoni* infection status: (1) no infection as the reference group; (2) *S. mansoni* mono-infection; and (3) *S. mansoni* co-infection with other parasitic species.

The morbidity-related parameters (the dependent variables) were regressed on the *S. mansoni* infection status (the independent variable) using two-level logistic regressions. Specifically, two-level logistic regression was done for each of the seven morbidity-related parameters, including self-rated health (poor vs good), anaemia (yes vs no), underweight (yes vs no), abdominal pain (yes vs no), pain score (> 2 vs ≤ 2), symptom score (> 4 vs ≤ 4) and healthcare use (yes vs no).

Three groups of analytical models based on *S. mansoni* diagnostic method were tested for each regression model. Model 1 pertains to *S. mansoni* infection defined as a positive Kato-Katz or a positive POC-CCA test including trace as positive (i.e., trace, 1 +, 2 + and 3 +). Model 2 refers to *S. mansoni* infection defined as a positive Kato-Katz or a positive POC-CCA test excluding trace positive-only cases (i.e., 1 +, 2 + and 3 +). Model 3 relates to *S. mansoni* infection defined as positive Kato-Katz test (excluding all POC-CCA positive-only cases).

For all analyses, effect estimates were considered as statistically significant if the two-sided *P-*value of the test statistic was < 0.05. Effect estimates were further considered as ‘indicating a trend’ if 0.05 ≤ *P* < 0.1. Analyses were performed using Stata version 14 (Stata Corporation, College Station, Texas, USA) and R Statistical Software version 3.6.2 (R Foundation; Vienna, Austria).

Of note, we used the following operational definitions for some key terms: poor handwashing practice meant lack of handwashing with soap; poor faecal handling practice was considered as disposal of faeces into a drain, bush, ditch, on the ground or garbage heap; and poor water storage meant storage of water in uncovered containers.

## Results

### Characteristics of the study participants

*Sociodemographic characteristics* Overall, 1019 adults were invited to participate in this study as we assumed that the final sample size would be reduced due to non-response and missing data. Among them, 118 were excluded from the analyses for missing data. Hence, final analyses included 901 individuals who had complete parasitological and questionnaire data. Table 1 shows characteristics of the included study participants. The median age of the participants was 41 years (range 18–90 years). Females represented 48.4% of the participants. There was an equal representation of semi-urban (50.1%) and rural (49.9%) dwellers. Most of the participants were farmers (54.1%). Slightly less than half (45%) of the participants had no formal education.

**Table 1:** Summary of participants’ characteristics of a cross-sectional survey carried out in the region of Taabo, south-central part of Côte d’Ivoire in April and May 2017

Group	Characteristic	Number (%)
	All	901 (100.0)
Sociodemographic factors	Age [years; median (IQR)]	41 (18)
	Age: 18–41 years	477 (52.9)
	Females	436 (48.4)
	No formal education	405 (45.0)
	Lowest wealth tertile	309 (34.3)
	Farmers	487 (54.1)
	Area: Taabo-Cité (semi-urban)	451 (50.1)
	Area: Amani-Ménou (rural)	222 (24.6)
	Area: Tokohiri (rural)	228 (25.3)
	Rural area	450 (49.9)
Hygiene-related factors	No toilet in household	426 (47.3)
	Use of surface water	201 (22.3)
	Disposal of waste in the environment	799 (88.7)
	Disposal of toilet water in the open	645 (71.6)
	Poor handwashing practice	6 (0.7)
	Poor water storage	115 (12.8)
	Poor faecal handling	446 (49.5)
	Poor hygiene score [median (IQR)]^a^	3 (2)
	Poor hygiene score > 3	371 (41.2)
Health-related factors	Fatigue	701 (77.8)
	Nausea or vomiting	154 (17.1)
	Fever	374 (41.5)
	Diarrhea	362 (40.2)
	Blood in the stool	169 (18.8)
	Blood in the urine	6 (0.7)
	General body pain	736 (81.7)
	General body pain disturbance	450 (61.1)
	Abdominal pain	346 (38.4)
	Use of pain medication	238 (26.4)
	Pain score [median (IQR)]^b^	2 (2)
	Pain score > 2	329 (36.5)
	Symptom score [median (IQR)]^c^	4 (3)
	Symptom score > 4	369 (41.0)
	Poor self-rated general health	43 (4.8)
	Use of conventional healthcare	697 (77.4)
	Hemoglobin, Hb [g/dl; median (IQR)]	13.3 (2.2)
	Anaemia	185 (20.5)
	Body mass index, BMI [kg/m^2^; median (IQR)]	22.7 (4.8)
	Underweight	69 (7.7)

All estimates are counts and proportion unless stated otherwise; *IQR* interquartile range

^a^Combines no toilet in household, use of surface water, disposal of household waste in nature or toilet water in the open and poor handwashing, fecal handling or water storage practices

^b^Combines general body pain, pain disturbance, abdominal pain and use of pain medication

^c^Combines pain score, fatigue, fever, nausea or vomiting, diarrhoea, blood in the stool and blood in the urine

*Hygiene-related characteristics* Among the included participants, 47.3% reported having no toilet in their house, and 22.3% reported use of surface water. The majority disposed their household waste in the environment (88.7%) and toilet water in the open (71.6%). Only 0.7% of individuals had poor handwashing practice. The proportion of individuals storing their water in unsanitary conditions was 12.8%. Almost half of participants (49.5%) had a poor faecal handling practice. Overall, the median score for poor hygiene was 3 (out of 7), and 41.2% of participants had a poor hygiene score > 3 (Table 1).

*Health-related characteristics* General body pain and pain disturbance were reported by 81.7% and 61.1% of participants, respectively. The proportion of people reporting abdominal pain was 38.4%, while that of individuals reporting use of pain medication was 26.4%. A median pain score of 2 (out of 4) was obtained, and 36.5% of the study population had a pain score > 2.

Among the other investigated symptoms, fatigue was often reported (77.8%). Only 18.8% and 0.7% of the participants reported blood in stool and blood in urine, respectively. Median score of symptoms reported by participants was 4 (out of 10) and 40.1% of people had a symptom score > 4. One-fifth of participants (20.5%) were anaemic, whereas 7.7% were underweight. More than three-quarters (77.4%) of the participants reported regular use of conventional healthcare services, whereas 4.8% of the participants reported poor general health (Table 1).

### Parasitic infections

Among the 901 participants, the most frequent infection diagnosed was *S. mansoni* with an overall prevalence of 23.2% (*n* = 209) when considering positive results from the Kato-Katz thick smear and the POC-CCA urine cassette test. The prevalence of *S. haematobium* infection was low (1.0%), giving a combined schistosomiasis prevalence of 24.0%. *S. mansoni* cases were 81.3% (*n* = 170) mono-infection and 18.7% (*n* = 39) co-infections with either *Plasmodium*, *S. haematobium* or hookworm. Overall, there were 96 participants (10.6%) with other infections, but without *S. mansoni*.

Due to the small number of *S. haematobium* cases, further analyses focused on *S. mansoni*. The distribution of *S. mansoni* infection within the study population is summarized in Table 2. Infection was significantly higher in younger (≤ 41 years) than in older (> 41 years) adults (30.0% vs 15.6%, *P* < 0.001). There was no significant difference (*P* > 0.05) in *S. mansoni* infection with regard to sex and educational attainment. *S. mansoni* infection prevalence varied considerably with the socioeconomic status, with the highest prevalence observed in the lowest tertile compared to the upper wealth tertiles (30.7% vs 19.3%, *P* < 0.001). Individuals involved in farming activities were more likely to be infected than those with non-farming occupation (25.7% vs 20.3%, *P* = 0.05). With regard to residency, there was no significant difference between the semi-urban and combined rural settings (*P* > 0.05). However, within the rural settings, prevalence of *S. mansoni* was significantly higher in Tokohiri compared to Amani-Ménou (29.4% vs 19.8%, *P* = 0.04).

**Table 2:** Prevalence of *Schistosoma mansoni* infection according to the sociodemographic factors

Sociodemographic variables	Examined	Prevalence (%)	*P*-value*
Total	901	23.2	–
Age ≤ 41 years	477	30.0	
Age > 41 years	424	15.6	3.10^–7^
Females	436	21.6	
Males	465	24.7	0.25
No formal education	405	23.9	
Formal education	496	22.6	0.62
Upper wealth tertiles	592	19.3	
Lowest wealth tertile	309	30.7	10^–4^
Non-farmers	414	20.3	
Farmers	487	25.7	0.05
Area: Taabo-Cité	451	21.7	
Area: Amani-Ménou	222	19.8^a^	
Area: Tokohiri	228	29.4^b^	0.04
Rural area	450	24.7	

*P-value: Probability value; Difference in infection prevalence between subgroups of the study sample was tested using the Pearson’s *χ*^2^; Difference between a and b is statistically significant (*P*-value < 0.05)

### Risk factors associated with *S. mansoni* mono-infection and co-infection with other species

The bivariate logistic regression model analysis results show the risk factors associated with *S. mansoni* infection status according to the diagnostic method (Table 3). Among adults of age > 41 years (vs age ≤ 41 years), *S. mansoni* mono- (all models) and co-infections (model 1) were less likely to be observed, when compared to no infection (*P* < 0.05). Belonging to the lowest wealth tertile (vs upper wealth tertile) increased the odds of both *S. mansoni* mono- and co-infections (all models) in the studied participants (*P* < 0.05). Farmers (vs non farmers) had higher odds of co-infections (model 1 and 2) (*P* < 0.05). Poor hygiene (score > 3 vs score ≤ 3) increased the odds of mono-infection (model 1) and co-infections regardless of the model (*P* < 0.05). Living in the semi-urban area (vs combined rural area) significantly reduced the odds of mono-infection (model 3) and co-infections (models 1 and 3) (*P* < 0.05). Formal education (vs. non formal education) reduced only the odds of co-infections (model 2) (*P* < 0.05). Males (vs females) tended to have higher odds of co-infections (model 1) (*P* < 0.1). The multivariate logistic regression analysis of the independent variables is detailed in Table 4. Age > 41 years, lowest wealth tertile and poor hygiene (score > 3) were still significantly associated with *S. mansoni* infection status (*P* < 0.05).

**Table 3:** Bivariate associations between *Schistosoma mansoni* infection status and sociodemographic and hygiene related factors according to the diagnostic method

Variables	Model 1 c*OR* (95% *CI*)			Model 2 c*OR* (95% *CI*)			Model 3 c*OR* (95% *CI*)		
	No infection	*S. mansoni* only	*S. mansoni* co-infection	No infection	*S. mansoni* only	*S. mansoni* co-infection	No infection	*S. mansoni* only	*S. mansoni* co-infection
*N* (%)	596 (74.0)	170 (21.1)	39 (4.9)	596 (85.8)	78 (11.2)	21 (3.0)	596 (95.2)	22 (3.5)	8 (1.3)
Age > 41	Ref	0.40 (0.28–0.60)***	0.39 (0.19–0.78)**	Ref	0.28 (0.16–0.48)***	0.63 (0.25–1.59)	Ref	0.19 (0.06–0.58)**	0.52 (0.12–2.20)
Males	Ref	1.12 (0.79–1.57)	1.77 (0.90–3.48)*	Ref	1.16 (0.72–1.86)	1.70 (0.66–4.39)	Ref	1.74 (0.72–4.21)	1.66 (0.39–6.99)
Formal education	Ref	0.98 (0.70–1.38)	0.60 (0.31–1.15)	Ref	1.31 (0.80–2.13)	0.36 (0.13–0.95)**	Ref	0.93 (0.39–2.18)	2.32 (0.46–11.61)
Lowest wealth tertile	Ref	1.89 (1.33–2.69)***	3.69 (1.90–7.17)***	Ref	1.88 (1.16–3.06)**	7.19 (2.54–20.30)***	Ref	3.08 (1.31–7.28)**	4.28 (1.01–18.14)**
Farmers	Ref	1.28 (0.91–1.80)	2.86 (1.37–5.98)**	Ref	1.21 (0.75–1.95)	5.26 (1.52–18.26)**	Ref	2.11 (0.85–5.26)	1.64 (0.39–6.95)
Semi-urban area	Ref	0.89 (0.63–1.25)	0.51 (0.26–1.00)**	Ref	0.86 (0.54–1.39)	0.33 (0.12–0.91)**	Ref	0.34 (0.13–0.89)**	0.30 (0.06–1.52)
Poor hygiene score > 3	Ref	1.52 (1.07–2.14)**	4.44 (2.17–9.11)**	Ref	1.42 (0.88–2.29)	9.31 (2.68–32.36)**	Ref	1.75 (0.74–4.10)	5.24 (1.05–26.22)**

Estimates represent increase or decrease in odds of *S. mansoni* only infection or *S. mansoni* co-infection compared to no infection. All estimates were derived from bivariate multinomial logistic regression models including each independent variable at a time. Poor hygiene score combines lack of household toilet, use of surface water, disposal of household waste or toilet water in nature or open and poor handwashing, faecal handling or water storage practices. Median (IQR) of poor hygiene score is 3 (2)

*IQR* interquartile range, *cOR* crude odds ratio, *CI* confidence intervals, *Ref.* reference

^‡^*S. mansoni* co-infection with either *Plasmodium*, *S. haematobium* or hookworm

*P-value between 0.05 and 0.1; **P-value < 0.05; ***P-value < 0.001

Model 1: All participants (*n* = 805); *S. mansoni* defined as positive Kato-Katz or point-of-care circulating cathodic antigen (POC-CCA);

Model 2: *n* = 695; *S. mansoni* defined as positive Kato-Katz or POC-CCA test (excluding trace positive-only cases);

Model 3: *n* = 626; *S. mansoni* defined as positive Kato-Katz test (excluding all POC-CCA positive-only cases)

**Table 4:** Multivariate logistic regression analysis of factors associated with *Schistosoma mansoni* infection status according to the diagnostic method

Sociodemographic variables	Model 1 a*OR* (95% *CI*)			Model 2 aOR (95% *CI*)			Model 3 a*OR* (95% *CI*)		
	No infection	*S. mansoni* only	*S. mansoni* co-infection^‡^	No infection	*S. mansoni* only	*S. mansoni* co-infection^‡^	No infection	*S. mansoni* only	*S. mansoni* co-infection^‡^
N (%)	596 (74.0)	170 (21.1)	39 (4.9)	596 (85.8)	78 (11.2)	21 (3.0)	596 (95.2)	22 (3.5)	8 (1.3)
Age > 41	Ref	0.39 (0.27–0.57)***	0.35 (0.17–0.69)**	Ref	0.27 (0.17–0.48)***	0.55 (0.21–1.42)	Ref	0.18 (0.06–0.52)**	0.64 (0.19–2.16)
Males	Ref	1.13 (0.78–1.63)	1.96 (0.94–4.11)*	Ref	1.06 (0.63–1.77)	1.94 (0.72–5.29)	Ref	1.71 (0.64–4.64)	1.42 (0.31–6.56)
Formal education	Ref	0.90 (0.60–1.33)	0.57 (0.29–1.13)	Ref	1.16 (0.66–2.03)	0.40 (0.14–1.10)*	Ref	0.82 (0.29–2.34)	2.63 (0.44–15.79)
Lowest wealth tertile	Ref	2.03 (1.23–3.35)***	1.82 (0.71–4.65)**	Ref	2.10 (1.10–3.99)**	2.82 (0.83–9.55)*	Ref	2.39 (0.65–8.35)	2.60 (0.27–24.87)
Farmers	Ref	1.06 (0.67–1.67)	1.29 (0.43–3.84)	Ref	1.10 (0.60–2.01)	1.40 (0.23–8.51)	Ref	1.13 (0.37–3.49)	0.54 (0.04–6.69)
Semi-urban area	Ref	1.44 (0.94–2.20)*	1.55 (0.60–4.02)	Ref	1.33 (0.73–2.45)	1.66 (0.54–5.10)	Ref	0.48 (0.18–1.33)	0.63 (0.15–2.67)
Poor hygiene score > 3	Ref	1.11 (0.71–1.74)	3.10 (1.22–7.83)**	Ref	1.02 (0.56–1.85)	4.79 (1.26–18.16)**	Ref	0.69 (0.24–2.01)	3.59 (1.00–12.90)**

Estimates represent increase or decrease in odds of *S. mansoni* only infection or *S. mansoni* co-infection compared to no infection. All estimates were derived from multivariable multinomial logistic regression models including all sociodemographic variables. Poor hygiene score combines lack of household toilet, use of surface water, disposal of household waste or toilet water in nature or open and poor handwashing, faecal handling or water storage practices. Median (IQR) of poor hygiene score is 3 (2)

*IQR* interquartile range, *aOR* adjusted odds ratio, *CI* confidence intervals, *Ref.* reference

^‡^*S. mansoni* co-infection with either *Plasmodium*, *S. haematobium* or hookworm;

*P-value between 0.05 and 0.1; **P-value < 0.05; ***P-value < 0.001

Model 1: All participants (*n* = 805); *S. mansoni* defined as positive Kato-Katz or point-of-care circulating cathodic antigen (POC-CCA);

Model 2: *n* = 695; *S. mansoni* defined as positive Kato-Katz or POC-CCA test (excluding trace positive-only cases);

Model 3: *n* = 626; *S. mansoni* defined as positive Kato-Katz test (excluding all POC-CCA positive-only cases)

### Association of *S. mansoni* mono-infection and co-infection with morbidity parameters

As shown in Table 5, participants with *S. mansoni* co-infections were the most likely to report poor health (models 2 and 3) and non-use of healthcare (model 2), when compared to participants with no infection (*P* < 0.05). However, only participants with *S. mansoni* mono-infection had higher odds of reporting pain and general symptoms (model 3), compared to participants with no infection (*P* < 0.05). There was a trend for higher likelihood of underweight among participants with co-infections (model 2) and abdominal pain among individuals with *S. mansoni* mono-infection (models 2 and 3) (*P* < 0.1), but this did not reach significance. No association was found between anaemia and *S. mansoni* infection status (*P* > 0.05).

**Table 5:** Association between *Schistosoma mansoni* infection status and health-related outcomes according to the diagnostic method

Health-related variables	Model 1 *OR* (95% *CI*)			Model 2 *OR* (95% *CI*)			Model 3 *OR* (95% *CI*)		
	No infection	*S. mansoni* only	*S. mansoni* co-infection^‡^	No infection	*S. mansoni* only	*S. mansoni* co-infection^‡^	No infection	*S. mansoni* only	*S. mansoni* co-infection^‡^
N (%)	596 (74.0)	170 (21.1)	39 (4.9)	596 (85.8)	78 (11.2)	21 (3.0)	596 (95.2)	22 (3.5)	8 (1.3)
Self-rated poor health	Ref	1.84 (0.84–4.03)	2.16 (0.57–8.27)	Ref	0.78 (0.17–3.53)	4.59 (1.06–19.92) **	Ref	NA	8.35 (1.24–56.32) **
Healthcare use	Ref	1.11 (0.72–1.72)	0.55 (0.27–1.13) *	Ref	1.12 (0.62–2.03)	0.35 (0.14–0.88) **	Ref	1.12 (0.39–3.22)	0.27 (0.06–1.18) *
Anaemia	Ref	0.84 (0.52–1.33)	0.47 (0.16–1.39)	Ref	0.61 (0.30–1.24)	0.18 (0.02–1.42)	Ref	0.22 (0.03–1.68)	NA
Underweight	Ref	0.61 (0.29–1.30	1.64 (0.63–4.29)	Ref	0.96 (0.39–2.38)	2.68 (0.90–8.02) *	Ref	1.58 (0.43–5.77)	1.52 (0.22–13.09)
Abdominal pain	Ref	1.00 (0.69–1.45)	0.89 (0.44–1.80)	Ref	1.52 (0.92–2.52) *	1.11 (0.44–2.81)	Ref	2.32 (0.91–5.92) *	0.37 (0.07–1.99)
Pain score > 2	Ref	1.03 (0.71–1.49)	0.87 (0.42–1.78)	Ref	1.29 (0.78–2.14)	1.46 (0.68–3.64)	Ref	2.54 (1.03–6.28) **	0.94 (0.21–4.14)
Symptom score > 4	Ref	0.84 (0.58–1.22)	0.61 (0.29–1.26)	Ref	1.14 (0.69–1.88)	1.08 (0.43–2.73)	Ref	3.19 (1.18–8.67) **	0.64 (0.14–2.92)

Estimates represent increase or decrease in odds of each outcome in *S. mansoni* only positive and *S. mansoni* co-infection compared to no infection. All estimates were derived from multivariable logistic regression models (one for each health-related outcome) containing the *S. mansoni* infection status and hygiene variables. Pain score included general body pain, pain severity, abdominal pain and pain medication. Median (IQR) of pain score was 2 (2). Symptom score included general body pain, pain severity, abdominal pain, pain medication, fatigue, blood in stool, blood in the urine, fever, nausea or vomiting and diarrhoea. Median (IQR) of symptom score was 4 (3). *n.a*. not applicable due to absence of self-rated poor health or anaemia in this group

*IQR* interquartile range, *OR* odds ratio, *CI* confidence intervals, *Ref.* reference, *NA* not available

^‡^*S. mansoni* co-infection with either *Plasmodium*, *S. haematobium* or hookworm;

*P-value between 0.05 and 0.1; **P-value < 0.05

Model 1: All participants (*n* = 805); *S. mansoni* defined as positive Kato-Katz or point-of-care circulating cathodic antigen (POC-CCA);

Model 2: *n* = 695; *S. mansoni* defined as positive Kato-Katz or POC-CCA test (excluding trace positive-only cases);

Model 3: *n* = 626; *S. mansoni* defined as positive Kato-Katz test (excluding all POC-CCA positive-only cases)

## Discussion

Our results confirmed the presence of both *S. mansoni* and *S. haematobium* among adults in the Taabo HDSS in the south-central part of Côte d’Ivoire, with the former being the predominant species. In agreement with prior observations in the study area [11, 15], the prevalence of *S. haematobium* was low, confirming a strong decrease in the prevalence of urogenital schistosomiasis, which heavily affected school-aged children in the mid-1990s [12]. The relatively high prevalence of *S. mansoni* infection in adults observed in our study suggests an increase in the level of endemicity of intestinal schistosomiasis in the Taabo HDSS. The increase of *S. mansoni* might be governed by social-ecological conditions. Yet, the diagnostic methods used for case identification is also worth mentioning. Indeed, in the present study, a composite measure for detection of *S. mansoni* infection was employed, consisting of the widely used Kato-Katz technique, coupled with a much more sensitive POC-CCA urine cassette test [25].

We observed a higher prevalence of *S. mansoni* infection in younger adults (18–41 years) compared to their older counterparts (aged 41 years and above), which might be explained by difference in behaviour. Young adults are more mobile and more engaged in farming activities that involve regular contacts with infested water bodies, which put them at a higher risk of *S. mansoni* infection than the older age group. This is particularly concordant in the village of Tokohiri, where most of people regularly cross the Bandama River to have access to their farms or visit their relatives living in hamlets at the opposite side of the river. Thus, the highest prevalence observed in this village may be attributed to the frequent contacts of residents with unprotected open freshwater that may contain infected intermediate host snails.

In line with previous investigations [26, 27], the prevalence of *S. mansoni* infection was similar among males and females. However, some studies have reported a significant sex difference, due to social or cultural considerations that can prevent women from having access to water bodies [28, 29]. In the current study area of south-central Côte d’Ivoire, women equally participated in outdoor water-related activities. Similarly, participants with formal and non-formal education were equally exposed to *S. mansoni* infection, which corroborates findings from Tanzania [30].

Low socioeconomic status was associated with higher odds of *S. mansoni* mono- and co-infections, suggesting that adults belonging to the poorest group constitute an important target for control of schistosomiasis in this setting. Poor hygiene was identified as a risk factor for co-infections. Indeed, in the study area, adults exhibited risky behaviours. For example, almost half and 88.7% of the individuals surveyed reported having no toilet at home, and hence, they disposed waste in the environment, thus potentially contaminating the environment with human and animal faecal pathogens [31–33]. As shown by Hürlimann et al. [34], including WASH as part of integrated control of schistosomiasis and other helminth and intestinal protozoa infections could be an effective approach to improve people’s health and wellbeing in the study area.

The tendency for lower odds of healthcare use among participants with co-infections is not surprising. In many areas of Africa, several infectious diseases coexist [35–37]. Hence, it is conceivable that people who do not seek treatment could accumulate the infections. An effective control strategy is regular mass administration of anthelminthic drugs outside the health care facilities (as recommended by WHO) and health education for prompt treatment of infections when symptoms occur.

Individuals with co-infections were more likely to rate their health as poor compared to their uninfected counterparts. A possible explanation of this association is that, unlike people harbouring no or showing a single infection, people exposed to two or more infectious agents may develop more episodes of illness or acute diseases that affect permanently their wellbeing. This result is consistent with another study conducted in South Africa, where infectious diseases have been found to be associated with self-rated poor health compared with absence of infection [38].

Abdominal pain is one of the common symptoms described for intestinal schistosomiasis [39, 40]. Other common manifestations of intestinal schistosomiasis include diarrhoea, bloody stool, fever, nausea, vomiting, chills, weakness, headache, anorexia and general malaise [41, 42]. In our study area, it is understandable that many of the symptoms used to determine the symptom score were found among *S. mansoni*-infected individuals. It is important to note that those symptoms are often nonspecific and may be caused by other helminth infections or malaria [43]. Given this, an association between co-infections and higher pain and symptom scores was also expected. However, a lack of association was observed. Pain and symptom scores were probably not reliable measurements of *S. mansoni* infection status among adults. Multimorbidity particularly due to chronic diseases (such as cardiovascular diseases, arthritis and other neuromuscular diseases) may have had an influence on participants’ pain and symptom responses relatively to their health outcomes [44].

In line with previous studies from Uganda [45], there was no evidence of the effect of *S. mansoni* infection status on anaemia. Anaemia is known to be multifactorial. Among adults, poor nutrition, for example, may be considered as a potential contributor [46, 47], which was, however, not assessed in our study.

### Strengths and limitations

Strengths of the present study were that a comprehensive approach was used in investigating the correlates of *Schistosoma* infection, including concurrent infections as well as factors related to clinical morbidity, in the same adult population. The well-characterized CoDuBu study allowed the investigation of these questions in a detailed manner. Two different methods for the diagnosis of *S. mansoni* (i.e., Kato-Katz thick smear and POC-CCA urine cassette test) were employed to limit bias of misclassifications. The Kato-Katz thick smear technique is the recommended method for *S. mansoni* diagnosis. However, it has a low sensitivity, especially among lightly infected individuals [48]. The POC-CCA has the potential to provide sensitive and accurate results [25, 49, 50], with the potential bottleneck of “trace” result interpretation [51]. Different definitions of infection were included, which strengthens the fidelity of our results.

The study is limited by its cross-sectional nature, which precludes causal inferences. Given the very low prevalence of Kato-Katz positive cases, associations with infection intensity could not be investigated. However, the findings remain valid and call for future longitudinal research on adults, incorporating a parallel investigation in children (preschool- and school-aged), for improved understanding of the disease dynamics across a broader age range. It is also acknowledged that the study area faces a high burden from several non-communicable diseases (NCDs), such as hypertension and diabetes [52, 53]. Hence, additional studies are warranted to investigate the concomitance of schistosomiasis and NCDs. This will help fill the data gap in areas of potential links between infectious diseases and NCDs and shed new light on their respective determinants.

## Conclusions

The present study provides important information that might be useful to enhance the control of schistosomiasis and other helminthiases in the Taabo region. First, although both urogenital and intestinal schistosomiasis were co-endemic, the predominant health problem relates to intestinal schistosomiasis. Second, younger adults (< 41 years), and those from low socioeconomic status or those practicing poor hygiene appeared to be more commonly infected by *S. mansoni* compared to their older and richer counterparts. Third, self-rated poor health, low use of healthcare, higher pain and symptom scores were the morbidity correlates of *S. mansoni* infection. Findings from the present study, taken together, show that adults represent a substantial reservoir of *S. mansoni*. Preventive chemotherapy with praziquantel targeting young adults, particularly those in the lowest wealth tertiles, every second year, coupled with specific interventions such as hygiene and health education, sanitation and improved access to clean water are necessary for the prevention and control of schistosomiasis in the Taabo HDSS.

## Data Availability

All data generated and analysed during this study are included in this published article.

## Abbreviations

BMI: Body mass index
CoDuBu: Côte d’Ivoire Dual Burden of Disease
EDTA: Ethylenediaminetetraacetic
HDSS: Health and demographic surveillance system
IQR: Interquartile range
NCD: Non-communicable diseases
*OR*: Odds ratio
POC-CCA: Point-of-care circulating cathodic antigen
SDG: Sustainable development goals
WASH: Water, sanitation and hygiene
WHO: World Health Organization

## References

[1] Diakité NR, N'Zi KG, Ouattara M, Coulibaly JT, Saric J, Yao PK (2018). Association of riverine prawns and intermediate host snails and correlation with human schistosomiasis in two river systems in south-eastern Côte d'Ivoire. Parasitology.

[2] Angora EK, Boissier J, Menan H, Rey O, Tuo K, Touré AO (2019). Prevalence and risk factors for schistosomiasis among schoolchildren in two settings of Côte d’Ivoire. Trop Med Infect Dis.

[3] Ouattara M, Diakité NR, Yao PK, Saric J, Coulibaly JT, Assaré RK (2021). Effectiveness of school-based preventive chemotherapy strategies for sustaining the control of schistosomiasis in Côte d’Ivoire: results of a 5-year cluster randomized trial. PLoS Negl Trop Dis.

[4] World Health Organization. Preventive chemotherapy in human helminthiasis: coordinated use of anthelminthic drugs in control interventions: a manual for health professionals and programme managers. Geneva: World Health Organization; 2006. Accessed 12 March 2020.

[5] Tchuem Tchuenté LA, N’Goran EK (2009). Schistosomiasis and soil-transmitted helminthiasis control in Cameroon and Côte d’Ivoire: implementing control on a limited budget. Parasitology.

[6] World Health Organization. Accelerating progress on HIV, tuberculosis, malaria, hepatitis and neglected tropical diseases. A new agenda for 2016–2030. Geneva: World Health Organization; 2015. Accessed 24 May 2020.

[7] World Health Organization. Ending the neglect to attain the Sustainable Development Goals: a road map for neglected tropical diseases 2021–2030. Geneva: World Health Organization; 2021. Accessed 08 May 2021.

[8] Opio CK, Kazibwe F, Ocama P, Rejani L, Belousova EN, Ajal P (2016). Profiling lifetime episodes of upper gastrointestinal bleeding among patients from rural Sub-Saharan Africa where *Schistosoma mansoni* is endemic. Pan Afr Med J.

[9] Gunda DW, Kilonzo SB, Manyiri PM, Peck RN, Mazigo HD (2020). Morbidity and mortality due to *Schistosoma mansoni* related periportal fibrosis: could early diagnosis of varices improve the outcome following available treatment modalities in Subsaharan Africa? A scoping review. Trop Med Infect Dis.

[10] Diakité NR, Winkler MS, Coulibaly JT, Guindo-Coulibaly N, Utzinger J, N’Goran EK (2017). Dynamics of freshwater snails and *Schistosoma* infection prevalence in schoolchildren during the construction and operation of a multipurpose dam in central Côte d’Ivoire. Infect Dis Poverty.

[11] Coulibaly G, Ouattara M, Dongo K, Hürlimann E, Bassa FK, Koné N (2018). Epidemiology of intestinal parasite infections in three departments of south-central Côte d’Ivoire before the implementation of a cluster-randomised trial. Parasite Epidemiol Control.

[12] N’Goran EK, Diabaté S, Utzinger J, Sellin B (1997). Changes in human schistosomiasis levels after the construction of two large hydroelectric dams in central Côte d’Ivoire. Bull World Health Organ.

[13] N’Goran EK, Utzinger J, N’Guessan AN, Müller I, Zamblé K, Lohourignon KL (2001). Reinfection with *Schistosoma haematobium* following school-based chemotherapy with praziquantel in four highly endemic villages in Côte d’Ivoire. Trop Med Int Health.

[14] Steinmann P, Keiser J, Bos R, Tanner M, Utzinger J (2006). Schistosomiasis and water resources development: systematic review, meta-analysis, and estimates of people at risk. Lancet Infect Dis.

[15] Fürst T, Ouattara M, Silué KD, N’Goran DN, Adiossan LG, Bogoch II (2013). Scope and limits of an anamnestic questionnaire in a control-induced low-endemicity helminthiasis setting in South-Central Côte d’Ivoire. PLoS ONE.

[16] Bassa KF, Ouattara M, Silué KD, Adiossan LG, Baikoro N, Koné S (2016). Epidemiology of malaria in the Taabo health and demographic surveillance system, south-central Côte d’Ivoire. Malar J.

[17] Koné S, Baikoro N, N’Guessan Y, Jaeger FN, Silué KD, Fürst T (2015). Health & demographic surveillance system profile: the Taabo health and demographic surveillance system. Côte d’Ivoire Int J Epidemiol.

[18] Eze IC, Essé C, Bassa FK, Koné S, Acka F, Yao L (2017). Côte d’Ivoire Dual Burden of Disease (CoDuBu): study protocol to investigate the co-occurrence of chronic infections and noncommunicable diseases in rural settings of epidemiological transition. JMIR Res Protoc..

[19] Garcia LS (2009). Practical guide to diagnostic Parasitology.

[20] Katz N, Chaves A, Pellegrino J (1972). A simple device for quantitative stool thick-smear technique in *Schistosomiasis mansoni*. Rev Inst Med Trop.

[21] Plouvier S, Leroy JC, Colette J (1975). A propos d’une technique simple de filtration des urines dans le diagnostic de la bilharziose urinaire en enquête de masse. Med Trop.

[22] Filmer D, Pritchett LH (2001). Estimating wealth effects without expenditure data-or tears: an application to educational enrollments in states of India. Demography.

[23] World Health Organization. Haemoglobin concentrations for the diagnosis of anaemia and assessment of severity. Vitamin and Mineral Nutrition Information Systems. Geneva: World Health Organization; 2011. Accessed 17 April 2020.

[24] World Health Organization. Obesity: preventing and managing the global epidemic. Report of a WHO consultation. WHO Tech Rep Ser 894. Geneva: World Health Organization; 2000. Accessed 17 April 2020. 11234459

[25] Bärenbold O, Garba A, Colley DG, Fleming FM, Haggag AA, Ramzy RMR (2018). Translating preventive chemotherapy prevalence thresholds for *Schistosoma mansoni* from the Kato-Katz technique into the point-of-care circulating cathodic antigen diagnostic test. PLoS Negl Trop Dis.

[26] Muhumuza S, Kitimbo G, Oryema-Lalobo M, Nuwaha F (2009). Association between socio-economic status and schistosomiasis infection in Jinja District. Uganda Trop Med Int Health.

[27] Bajiro M, Dana D, Ayana M, Emana D, Mekonnen Z, Zawdie B (2016). Prevalence of *Schistosoma mansoni* infection and the therapeutic efficacy of praziquantel among school children in Manna District, Jimma Zone, southwest Ethiopia. Parasites Vectors.

[28] Sady H, Al-Mekhlafi HM, Mahdy MA, Lim YA, Mahmud R, Surin J (2013). Prevalence and associated factors of schistosomiasis among children in Yemen: implications for an effective control programme. PLOS Negl Trop Dis.

[29] Dawaki S, Al-Mekhlafi HM, Ithoi I, Ibrahim J, Abdulsalam AM, Ahmed A, et al. Prevalence and risk factors of schistosomiasis among Hausa communities in Kano State, Nigeria. Rev Inst Med Trop. 2016; 58. 10.1590/S1678-9946201658054 PMC4964323 27410914

[30] Mazigo HD, Nuwaha F, Dunne DW, Kaatano GM, Angelo T, Kepha S (2017). *Schistosoma mansoni* infection and its related morbidity among adults living in selected villages of Mara region, North-Western Tanzania: a cross-sectional exploratory study. Korean J Parasitol.

[31] Ambesh P, Ambesh SP. Open defecation in India: a major health hazard and hurdle in infection control. J Clin Diagn Res. 2016; IL01–IL02. 10.7860/JCDR/2016/20723.8098 PMC5020240 27630866

[32] Samuel F (2016). Excreta-related infections and the role of latrines to control the transmission in Ethiopia. J Community Med Health Educ.

[33] Ahmed AS, Halabi Z, Antoun J (2019). The effect of the waste disposal crisis on the rates of hospitalization due to acute diarrheal illness in a middle-income country: retrospective chart review. Int J Infect Dis.

[34] Hürlimann E, Silué KD, Zouzou F, Ouattara M, Schmidlin T, Yapi RB (2018). Effect of an integrated intervention package of preventive chemotherapy, community-led total sanitation and health education on the incidence of helminth and intestinal protozoa infections in Côte d'Ivoire. Parasites Vectors.

[35] Erismann S, Diagbouga S, Odermatt P, Knoblauch AM, Gerold J, Shrestha A (2016). Prevalence of intestinal parasitic infections and associated risk factors among schoolchildren in the Plateau Central and Centre-Ouest regions of Burkina Faso. Parasites Vectors.

[36] Babamale OA, Ugbomoiko US, Heukelbach J (2018). High prevalence of *Plasmodium falciparum* and soil-transmitted helminth co-infections in a periurban community in Kwara State. Nigeria J Infect Public Health.

[37] Tuasha N, Hailemeskel E, Erko B, Petros B (2019). Comorbidity of intestinal helminthiases among malaria outpatients of Wondo Genet health centers, southern Ethiopia: implications for integrated control. BMC Infect Dis.

[38] Made F, Ntlebi V, Kootbodien T, Wilson K, Tlotleng N, Mathee A (2020). Illness, self-rated health and access to medical care among waste pickers in landfill sites in Johannesburg, South Africa. Int J Environ Res Public Health.

[39] Barsoum RS, Esmat G, El-Bazb T (2013). Human schistosomiasis: clinical perspective: review. J Adv Res.

[40] Shuja A, Guan J, Harris C, Alkhasawneh A, Malespin M, De Melo S (2018). Intestinal schistosomiasis: a rare cause of abdominal pain and weight loss. Cureus..

[41] Da Silva LC, Chieffi PP, Carrilho FJ (2005). *Schistosomiasis mansoni*—clinical features. Gastroenterol Hepatol.

[42] Verjee MA (2019). Schistosomiasis: still a cause of significant morbidity and mortality. Res Rep Trop Med.

[43] Rai N, Abraham J (2012). Different clinical features of malaria. Asian J Biomed Pharm Sci.

[44] Arokiasamy P, Uttamacharya U, Jain K, Biritwum BR, Yawson AE, Wu F (2015). The impact of multimorbidity on adult physical and mental health in low- and middle-income countries: what does the study on global ageing and adult health (SAGE) reveal?. BMC Med.

[45] Tukahebwa EM, Magnussen P, Madsen H, Kabatereine NB, Nuwaha F, Wilson S (2013). A very high infection intensity of *Schistosoma mansoni* in a Ugandan Lake Victoria fishing community is required for association with highly prevalent organ related morbidity. PLOS Negl Trop Dis.

[46] Dobner J, Kaser S (2018). Body mass index and the risk of infection—from underweight to obesity. Clin Microbiol Infect.

[47] Chaparro CM, Suchdev PS (2019). Anemia epidemiology, pathophysiology and etiology in low- and middle-income countries. Ann N Y Acad Sci.

[48] Berhe N, Medhin G, Erko B, Smith T, Gedamu S, Bereded D (2004). Variations in helminth faecal egg counts in Kato-Katz thick smears and their implications in assessing infection status with *Schistosoma mansoni*. Acta Trop.

[49] Coulibaly JT, Knopp S, N’Guessan NA, Silué KD, Furst T, Lohourignon LK (2011). Accuracy of urine circulating cathodic antigen (CCA) test for *Schistosoma mansoni* diagnosis in different settings of Côte d’Ivoire. PLoS Negl Trop Dis.

[50] Okoyo C, Simiyu E, Njenga SM, Mwandawiro C (2018). Comparing the performance of circulating cathodic antigen and Kato-Katz techniques in evaluating *Schistosoma mansoni* infection in areas with low prevalence in selected counties of Kenya: a cross-sectional study. BMC Public Health.

[51] Coelho PMZ, Siqueira LMV, Grenfell RFQ, Almeida NBF, Katz N, Almeida A (2016). Improvement of POC-CCA interpretation by using lyophilization of urine from patients with *Schistosoma mansoni* low worm burden: towards an elimination of doubts about the concept of trace. PLoS Negl Trop Dis.

[52] Eze IC, Bassa KF, Essé C, Koné S, Acka F, Laubhouet-Koffi V (2019). Epidemiological links between malaria parasitaemia and hypertension: findings from a population-based survey in rural Côte d'Ivoire. J Hypertens.

[53] Eze IC, Essé C, Bassa FK, Koné S, Acka F, Schindler C (2019). Asymptomatic *Plasmodium* infection and glycemic control in adults: results from a population-based survey in south-central Côte d’Ivoire. Diabetes Res Clin Pract.

